# Improvement in Random Noise for Pixel-Parallel Single-Slope ADC with Consideration of Flicker Noise Effect †

**DOI:** 10.3390/s25247565

**Published:** 2025-12-12

**Authors:** Masayuki Uno, Kwuang-Han Chang, Tsung-Hsun Tsai, Junichi Nakamura, Rimon Ikeno, Kazuya Mori, Ken Miyauchi, Toshiyuki Isozaki, Yi-Hsuan Lin, Sheng-Yeh Lai, Chih-Hao Lin, Wei-Fan Chou, Guang Yang, Song Chen, Chiao Liu

**Affiliations:** 1 Brillnics Japan Inc., 6-21-12 Minami-Oi, Shinagawa-ku, Tokyo 140-0013, Japan; nakamura.junichi@brillnics.com (J.N.); ikeno.rimon@brillnics.com (R.I.); mori.kazuya@brillnics.com (K.M.); miyauchi.ken@brillnics.com (K.M.); isozaki.toshiyuki@brillnics.com (T.I.); 2 Brillnics Inc., Zhubei City 302, Taiwan; kh.chang@brillnics.com (K.-H.C.); ethan.lin@brillnics.com (Y.-H.L.); sy.lai@brillnics.com (S.-Y.L.); howard.lin@brillnics.com (C.-H.L.); weifan.chou@brillnics.com (W.-F.C.); guang.yang@brillnics.com (G.Y.); 3 Meta Platforms Inc., Reality Labs, Redmond, WA 98052, USA; etsai@meta.com (T.-H.T.); song.chen@meta.com (S.C.); chiaoliu@meta.com (C.L.)

**Keywords:** pixel-parallel ADC, random noise, digital pixel sensor, CMOS image sensor, flicker noise, single-slope, correlated double sampling

## Abstract

**Highlights:**

**What are the main findings?**

- Limiting the noise bandwidth for low random noise is less effective if the main contributor to noise performance in digital-pixel sensors is flicker noise of the comparator’s input transistor.

**What is the implication of the main finding?**

- Low-noise digital-pixel sensor requires a low-flicker-noise device because of its pixel area limitation.

**Abstract:**

We propose and demonstrate a low-random-noise (RN) design for pixel-parallel single-slope ADCs (SS-ADCs), achieving 2.2 e-rms in a 3.24 µm pixel. In this paper, we discuss AC-based RN estimation with respect to the comparator bias current and a bandwidth-limiting capacitor in digital-pixel sensors (DPSs). RN is composed of thermal noise (TN) and flicker noise (FN), where FN can be a major contributor in DPSs because of its area limitation. We express the concise equation to estimate the FN/TN ratio, in which the FN characteristic is modulated by the correlated double sampling (CDS) operation. We also study the effective RN bandwidth, which increases due to the ramp slope transient effect and introduces a noise bandwidth (NBW) coefficient, to estimate the effective NBW. This study provides insights into the area arrangement of the small-pixel DPS design. A high-gain single-ended comparator is introduced to realize an area-efficient DPS without digital CDS (D-CDS). Noise analysis of its pixel design shows that FN becomes the main contributor, and further RN improvement by limiting NBW or D-CDS is not promising under these conditions.

## 1. Introduction

CDS was originally applied to suppress RN associated with a reset of the output floating diffusion (FD) in the CCD [1]. It has evolved to the D-CDS technique for a column-parallel SS-ADC in a CMOS image sensor (CIS) combined with analog-CDS (A-CDS) [2]. D-CDS suppresses not only FPN but also RN after A-CDS, and it suppresses the reset noise of the comparator auto-zero (AZ) operation after the suppression of pixel FD reset noise by A-CDS. Low noise of less than 1 e-_rms_ was reported by D-CDS [3]. The main contributor to low noise is the narrow NBW of a comparator in the column-parallel configuration and high conversion gain (HCG).

D-CDS is also applicable to the pixel-parallel ADC [4,5,6], and improved RN performances have been recently reported using a suitable NBW [7,8]. However, their RNs were still larger than those in the column-parallel configuration due to the small digital pixel area. In this report, the effectiveness of RN reduction with the D-CDS technique is investigated based on the DPS architecture reported in [9]. We found that D-CDS for RN suppression becomes less effective when the FN of the comparator’s input transistor is large. A low-RN design suitable for small pixels is implemented and demonstrated with consideration of FN.

## 2. Random Noise in Single-Slope ADC

**Figure 1:**
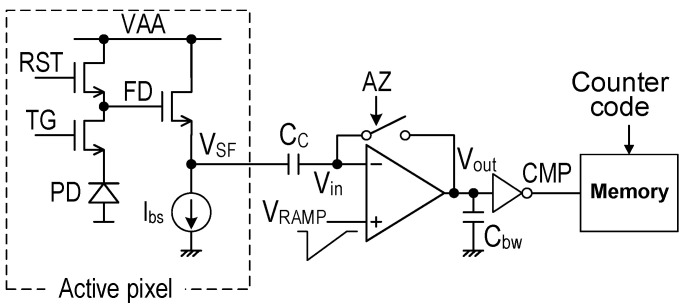
Simplified pixel-parallel SS-ADC circuit in [9]. The Active pixel has a constant current source of *I_bs_*.

A simplified circuit diagram of the pixel-parallel ADC circuit in [9] is shown in Figure 1. This circuit is basically the same as that of the conventional column-parallel SS-ADC configuration, where the pixel bias $I_{bs}$, comparator, and memory are implemented in each column. Thus, it is challenging to place them in a small pixel area. The output of the four-transistor active pixel is connected to the negative input of a differential amplifier through a coupling capacitor $C_{C}$, and the comparator output is connected to a load capacitor $C_{bw}$ and the inverter stage. Higher $C_{bw}$ reduces NBW and achieves low noise characteristics, as demonstrated in [10]. However, the maximum $C_{bw}$ size is restricted by the available device sizes and pixel size. As a result, the sizes of $C_{C}$ and $C_{bw}$ should be carefully considered in the pixel-parallel ADC configuration.

**Figure 2:**
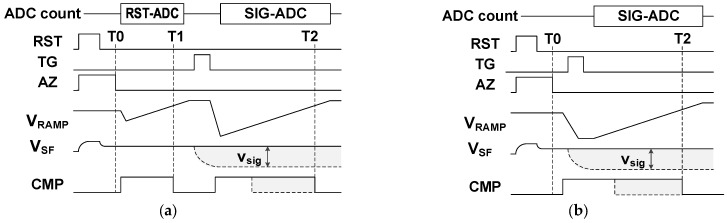
Simplified operation timing diagrams of SS-ADC: (**a**) D-CDS and (**b**) A-CDS operations.

SS-ADC operation timing diagrams are shown in Figure 2a for the D-CDS operation, and in Figure 2b for the operation where only A-CDS is performed, respectively. The pixel reset operation of RST = H to L induces reset noise at FD. This can be suppressed by the comparator auto-zero (AZ) operation through the feedback switch in the differential amplifier. The AZ operation of AZ = H to L leaves sampled noise in $C_{C}$ at T0. After the AZ operation, the first SS-ADC operation (RST-ADC) with the ramping signal V_RAMP_ is performed in the D-CDS. This is omitted in the A-CDS. After PD charge transfer with TG = H, the second (the first for the A-CDS) SS-ADC operation (SIG-ADC) is conducted. The sampled RN at T0 is converted to digital code as a kind of offset in both SS-ADC operations. The RN and residual fixed pattern noise (FPN) at T0 are subtracted in the D-CDS, while the amplifier RN at T1 is introduced again.

**Figure 3:**
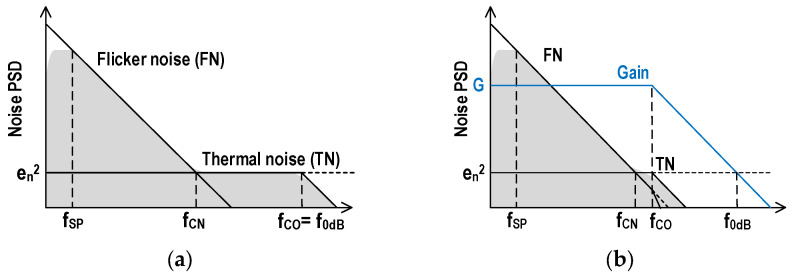
Conceptual RN PSD of the comparator in Figure 2 using input-referred TN PSD *e_n_*: RN PSD at (**a**) T0 under closed-loop condition; (**b**) T1 and T2 under open-loop condition. Blue line shows the frequency characteristic of amplifier gain. Noise voltage is calculated by the integration of gray block which becomes smaller in low-frequency region as well as high-frequency region.

Figure 3a,b show the conceptual input-referred RN power spectrum density (PSD) at T0 and T2, and this PSD is identical in both T1 and T2. The cut-off frequency of the amplifier $f_{CO}$ matches the unity gain frequency $f_{0dB}$ at T0 when the AZ operation is completed. It decreases at T2 when the comparator is in the open-loop condition with gain G. D-CDS works effectively in the column SS-ADC because lowering $f_{CO}$ reduces RN at T1 to a few times smaller than that at T0 because of the design flexibility for a suitable bias current and area arrangement of the comparator with $C_{bw}$. On the other hand, it should be carefully considered in DPS because they are restricted by pixel size and the number of pixels. We need to evaluate RN at T0 and T1 to make a decision whether D-CDS should be applied because D-CDS requires twice the memory area.

## 3. Random Noise Estimation

This section describes noise equations to estimate RN at T0 and T2 (T1). Section 3.1 shows noise equations of conventional AC-based noise analysis under the wide-sense-stationary (WSS) assumption [11]. In Section 3.2, an FN feature is considered. In Section 3.3, to consider RN under a nonstationary condition [11,12,13], the comparator delay time and effective NBW calculation are introduced.

### 3.1. Noise Analysis by Conventional AC-Based Noise Estimation

RN equations based on conventional AC-based noise analysis under the WSS assumption are shown in this section as a first-order noise study. It was reported in [9] that the comparator RN is the main noise contributor in the stacked DPS, which consists of two wafers: the CIS layer and ADC layer. This is because the FN of the active pixel source follower (SF) on the CIS layer is smaller than that of the comparator on the ADC layer. In addition, TN of the CIS layer can be suppressed by setting a suitable SF bias current ($I_{bs}$) and limiting the comparator bandwidth.

The input-referred RN of $V_{RN}$ at Vin in Figure 1 is written as Equation (1), assuming noise PSD in Figure 3. The first term inside the bracket is TN, and the second term is FN, where $e_{n}$ is the noise floor of TN and $f_{CN}$ is the corner frequency at which the noise PSD of FN matches that of TN. Using the cut-off frequency of $f_{CO}$ and the CDS frequency of $f_{SP}$, Equation (1) is transformed to Equation (2). In this equation, the coefficient of π/2 is included in the TN term for the equivalent NBW [14], but it is omitted in the FN calculation for simplicity (less impact). The constant b is an integrated value of the FN PSD below $f_{SP}$, which is calculated in Section 3.2. Two cases of $f_{SP}$, D-CDS and A-CDS, in Figure 2, are expressed as Equation (3).

(1) $${V_{RN}}^{2}={V_{TN}}^{2}+{V_{FN}}^{2}=\int \left\{{e_{n}}^{2}+\frac{f_{CN}}{f}{e_{n}}^{2}\right\}df$$

(2) $${V_{RN}}^{2}\;\approx \;{e_{n}}^{2}\left\{f_{CO}\frac{\pi }{2}+f_{CN}\left(\ln\frac{f_{CO}}{f_{SP}}+b\right)\right\}$$

(3) $$f_{SP}=1/(T_{2}-T_{1}):\mathrm{for}\;D-\mathrm{CDS}\;f_{SP}=1/(T_{2}\;-T_{0}):\mathrm{for}\;A-\mathrm{CDS}$$

Assuming a simple differential amplifier for the comparator input stage, $e_{n}$ is expressed by Equation (4). The cut-off frequency $f_{CO}$ is given by Equation (5). Two cases of the comparator condition, open-loop (T1 and T2) and closed-loop (T0), are indicated. Load capacitances are $C_{bw}$ in the former case, and $\;(C_{C}\;+\;C_{bw})$ in the latter case.

(4) $${e_{n}}^{2}=2\frac{4kT\gamma }{g_{mi}}(1\;+\;\frac{g_{mo}}{g_{mi}})$$

(5) $$f_{CO}=\frac{g_{mi}}{2\pi GC_{bw}}:\mathrm{at}\;T1,T2\;f_{CO}=\frac{g_{mi}}{2\pi (C_{C}+C_{bw})}:\mathrm{at}\;T0$$

TN is formulated as Equation (6) from Equations (4) and (5), where *k, T,* γ, $g_{mi}$, and $g_{mo}$ are the Boltzmann constant, absolute temperature, body-effect parameter, transconductance of the input MOS transistor, and that of the load MOS transistor, respectively.

(6) $${V_{TN}}^{2}=\frac{2\gamma kT}{G\cdot C_{bw}}\left(1+\frac{g_{mo}}{g_{mi}}\right):\mathrm{at}\;T1,T2\;{V_{TN}}^{2}=\frac{2\gamma kT}{C_{C}+C_{bw}}\left(1+\frac{g_{mo}}{g_{mi}}\right):\mathrm{at}\;T0$$

Equation (2) can be expressed as Equation (7). This equation enables us to estimate the FN effect easily because the second term inside the bracket shows the ratio of FN and TN.

(7) $${{{V_{RN}}^{2}=V}_{TN}}^{2}\left(1+\frac{{V_{FN}}^{2}}{{V_{TN}}^{2}}\right)\;\approx \;{V_{TN}}^{2}\left\{1+\frac{2}{\pi }\frac{f_{CN}}{f_{CO}}\left(\ln\frac{f_{CO}}{f_{SP}}+b\right)\right\}$$

It is convenient to use Equation (7) to estimate the effectiveness of limiting bandwidth for RN reduction. It can easily estimate the FN ratio by the second term. If it is relatively large (>0.5), the NBW limiting technique becomes inefficient because smaller $f_{CO}$ makes the second term large, even though ${V_{TN}}^{2}$ becomes small by limiting NBW.

### 3.2. Flicker Noise Feature

**Figure 4:**
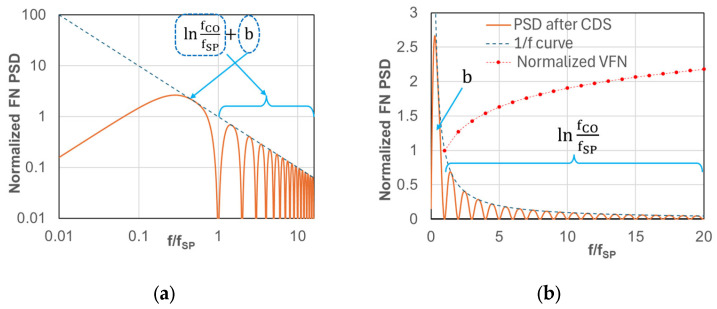
Normalized FN PSD in CDS operation shown in Figure 2 with 1/f curve as shown by dotted line: (**a**) log axis; (**b**) linear axis with additional curve of normalized *V_FN_*. Constant b is an integration value of $f<\;f_{SP}$, and $\ln f_{CO}/f_{SP}$ is that of $f>f_{SP}$.

The constant b in Equations (2) and (7) is the integration value of the FN PSD below $f_{SP}$ and is led by the transfer function of the circuit and its operation in general. Here, the CDS transfer function is considered to re-calculate the constant b after CDS. Figure 4 shows the FN PSD modulated by the CDS operation. Both logarithmic and linear scale plots are presented in Figure 4a and Figure 4b, respectively. The dotted blue line shows a 1/f function, and the frequency on the X axis is normalized by $f_{SP}$. It should be noted that $f_{SP}$ by itself does not impact FN after CDS, and the ratio of $f_{CO}/f_{SP}$ only affects FN voltage. For example, in the case of $f_{SP}^{’}\;=\;f_{SP}/n$, the PSD in the $f_{SP}^{’}$ case is *n* times larger than that in the $f_{SP}$ case, resulting in a constant integral value. The red plots in Figure 4b indicate the normalized FN voltage of $f_{CO}\;=\;mf_{SP}\;(m\;=\;1,2\cdots )$ by the constant b, which is an integration value from f = 0 to $f_{SP}$. After fitting it to the curve of $\ln f_{CO}/f_{SP}\;+\;b$, the constant b is calculated to be b≈2.5. It is a relatively large value compared to $\ln f_{CO}/f_{SP}$. Referring to the FN voltage ${V_{FN}}^{2}$ under the condition of $f_{CO}=f_{SP}$, it increases by a factor of 2 at $f_{CO}\;\approx \;12\;f_{SP}$ and 3 at $f_{CO}\;\approx \;140{\;f}_{SP}$, and is a feature of FN after CDS. Lowering $f_{CO}$ (NBW) reduces the TN voltage $V_{TN}$ effectively, but it does not efficiently work for FN, because of the large value of the constant b.

**Figure 5:**
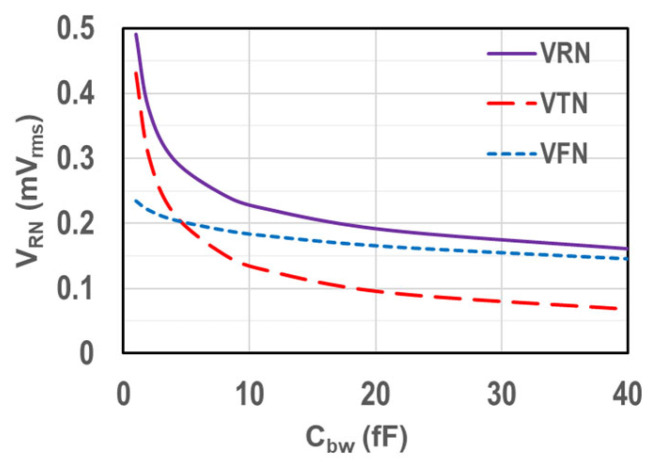
Example of RN calculation result at T1 (T2) showing C_bw_ dependency of TN and FN under the assumption of ${I_{bc}\;=\;20\;nA,\;G\;=\;100,\;g_{mo}/g_{mi}\;=\;0.5,\;f}_{SP}\;=\;20\;kHz,\;and\;f_{CN}\;=\;50\;kHz$.

An example of the calculated results of the $C_{bw}$ dependence of RN associated with TN and FN at T1 (T2) is shown in Figure 5 using Equation (7) with parameter values of ${I_{bc}\;=\;20\;nA,\;G\;=\;100,\;g_{mo}/g_{mi}\;=\;0.5,\;f}_{SP}\;=\;20\;kHz,\;and\;f_{CN}\;=\;50\;kHz$. RN decreases with $C_{bw}$ but exhibits a diminishing decrease once $C_{bw}$ becomes relatively large, especially when FN is the major contributor. RN at T0 approaches that at T1 when FN becomes the main contributor because of the lower $f_{CO}$ at T1, suggesting a regressed D-CDS effect on RN suppression. We also need to consider the effective NBW by the ramp slope at T1 under the nonstationary condition, which is described in Section 3.3.

FN is also expressed as Equation (8), where $C_{oxi}{,\;W}_{i}{,\;L}_{i},\;and\;K_{Fi}$ are the unit capacitance, width, length, and flicker coefficient of the input MOS transistor, respectively, and $C_{oxo}{,\;W}_{o}{,\;L}_{o},\;and\;K_{Fo}$ are those of the load MOS transistor, respectively. The coefficient ${{e_{n}}^{2}f}_{CN}$ includes effects of both input and load MOS transistors, and it is available through SPICE AC simulation. This equation indicates that FN reduction requires a large transistor size or device improvement for smaller $K_{F}$.

(8) $${V_{FN}}^{2}={{e_{n}}^{2}f}_{CN}\left(\ln\frac{f_{CO}}{f_{SP}}+b\right)=\left(\frac{K_{Fi}}{C_{oxi}W_{i}L_{i}}+\frac{g_{mo}}{g_{mi}}\frac{K_{Fo}}{C_{oxo}W_{o}L_{o}}\right)\left(\ln\frac{f_{CO}}{f_{SP}}+b\right)$$

One of the challenges in DPS RN improvement is FN reduction because of the limited area for a comparator. Correlated multiple sampling (CMS) [15] is a candidate for FN reduction after considering an effective circuit scheme.

### 3.3. Delay Time Calculation to Compensate Effective Noise Bandwidth Under Nonstationary Condition

**Figure 6:**
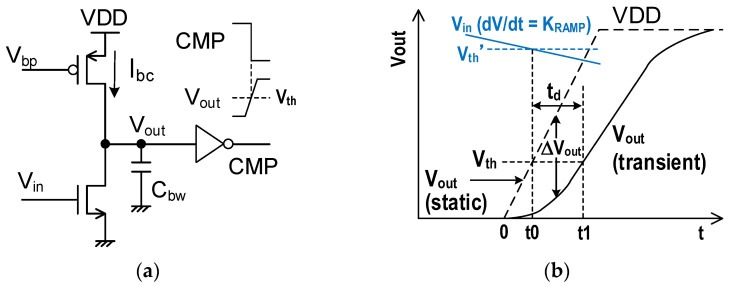
Constant-current inverter circuit and its V_out_ characteristic when the ramp signal with a slope of *K_RAMP_* is given at V_in_ (**a**) constant-current inverter circuit; (**b**)V_out_ waveforms to calculate delay time. The dotted line is an ideal curve without delay, and the solid line is a transient response curve. Solid blue line is a conceptual ramp signal input which meets the threshold voltage at t_0_.

Some papers show that the actual effective NBW of a comparator becomes higher than that in the WSS assumption [11,12,13]. The effective NBW is calculated using the comparator response time [11], which relates to input ramp slope and delay time. In this study, the delay time variation using the input ramp slope is calculated in a constant-current inverter, as shown in Figure 6a. It is composed of an input nMOS transistor and a load pMOS transistor as a constant current source ($I_{bc}$). It is assumed that the comparator output of CMP flips when $V_{out}$ becomes $V_{th}$, where $V_{th}$ is the threshold voltage at which CMP flips. Figure 6b shows its $V_{out}$ characteristic with delay (transient, solid line) and without (static, dashed line). It is assumed that a gain of $G\;=\;V_{out}/V_{in}$ is constant from $V_{out}\;=\;0$ to VDD for first-order estimation. The point where $V_{out}$ starts rising is shown as t=0. After that, the inverter charges the load capacitance $C_{bw}$, and the delay time of $t_{d}$ is defined from the cross point on the dashed line ($t=t_{0}$) to that on the solid line ($t=t_{1}$) as $t_{d}\;=\;t_{1}\;-\;t_{0}$_._

As the charging current of $C_{bw}$ is $g_{mi}∆V_{in}\left(t\right)$, which can be written as $g_{mi}∆V_{out}\left(t\right)/G$, the transient waveform of $V_{out}\left(t\right)$ is expressed as Equation (9) using the amplifier time constant $\tau_{O}$, which is defined as Equation (10). $∆V_{out}\left(t\right)$ is the difference between $V_{out}$ without delay (dashed line) and with (solid line), and it is expressed as Equation (11) using the ramp slope $K_{RAMP}$ at $V_{in}$.

(9) $$V_{out}\left(t\right)=\int_{0}^{t}\frac{g_{mi}}{C_{bw}}∆V_{in}\left(t\right)\mathrm{d}t=\frac{1}{\tau_{O}}\int_{0}^{t}∆V_{out}(t)\mathrm{d}t$$

(10) $$\tau_{O}=\frac{C_{bw}G}{g_{mi}}$$

(11) $$∆V_{out}\left(t\right)=K_{RAMP}G\cdot t-V_{out}\left(t\right)=K_{RAMP}G\cdot t-\frac{1}{\tau_{O}}\int_{0}^{t}∆V_{out}(t)\mathrm{d}t$$

Solving Equation (11) on $∆V_{out}\left(t\right)$ gives Equations (12) and (13).

(12) $$∆V_{out}\left(t\right)=K_{RAMP}G\cdot \tau_{O}\left\{1-exp\left(-\frac{t}{\tau_{O}}\right)\right\}$$

(13) $$V_{out}\left(t\right)=K_{RAMP}G\left\{t-\tau_{O}\left\{1-exp\left(-\frac{t}{\tau_{O}}\right)\right\}\right\}$$

Equation (13) gives the same result as reference [13], and it uses an approximation. However, numerical calculation is applied here to investigate the relationship between $t_{0}/\tau_{O}$ and $t_{d}/\tau_{O}$ from a steep-slope to slow-slope region. The crossing points of $t_{0}$ and $t_{1}$ are expressed as Equation (14) and Equation (15), respectively.

(14) $$t_{0}=\frac{V_{th}}{K_{RAMP}G}$$

(15) $$V_{out}\left(t1\right)=K_{RAMP}G\left\{t_{1}-\tau_{O}\left\{1-exp\left(-\frac{t_{1}}{\tau_{O}}\right)\right\}\right\}=V_{th}$$

Equations (14) and (15) lead to Equation (16) using $t_{1}\;=\;{t_{d}\;+\;t}_{0}$, and it is written as Equation (17).

(16) $$t_{d}=t_{1}-t_{0}=\tau_{O}\left\{1-exp\left(-\frac{t_{d}+t_{0}}{\tau_{O}}\right)\right\}$$

(17) $$\left(1-\frac{t_{d}}{\tau_{O}}\right)exp\left(\frac{t_{d}}{\tau_{O}}\right)=exp\left(-\frac{t_{0}}{\tau_{O}}\right)$$

**Figure 7:**
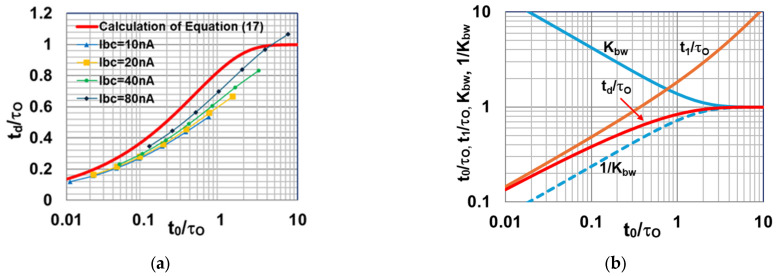
Calculation result of Equation (17) with SPICE simulation result and Equation (18): (**a**) calculated normalized delay time $t_{d}/\tau_{O}$ and simulation results in a simple current source inverter; (**b**) calculated effective NBW coefficient $K_{bw}$, $t_{d}/\tau_{O}$, and $t_{1}/\tau_{O}\;(=\;t_{d}/\tau_{O}\;+\;t_{0}/\tau_{O})$.

Equation (17) indicates that $t_{d}/\tau_{O}$ is calculated as a function of $t_{0}/\tau_{O}$. A numerical calculation result of the relationship between $t_{0}/\tau_{O}$ and $t_{d}/\tau_{O}$ is shown in Figure 7a as a thick red line. It indicates that a smaller $t_{0}/\tau_{O}$ with a steep ramp slope of $K_{RAMP}$ leads to the normalized delay time $t_{d}/\tau_{O}$ in a faster time. SPICE simulation results of the inverter circuits for four conditions of bias current are also plotted in Figure 7a. The simulation results roughly match the calculated result of $t_{d}/\tau_{O}$ in Equation (17). According to reference [11], the effective NBW (= NBW’) increases when the ramp slope is steep, and it is calculated with Equation (18) using the response time of $t_{1}\left(\;=t_{0}\;+\;t_{d}\right)$, where ${NBW}_{WSS}$ is the NBW under the WSS condition. Figure 7b shows the calculated $K_{bw}$. As $t_{0}/\tau_{O}$ increases, $K_{bw}$ approaches 1. This means that conventional RN estimation under the WSS assumption can be simply applied under the slow-ramp-slope condition of $t_{0}/\tau_{O}\;>\;2$. The value of $t_{0}/\tau_{O}$ is an important factor for SS-ADC operation, regarding it as the slope factor. It can easily estimate the delay time and the effective NBW as a first-order approximation. Its concept makes the comparator design on the SS-ADC easier.

(18) $$NBW^{\prime }=K_{bw}\cdot {NBW}_{WSS}=coth\left(\frac{t_{0}+t_{d}}{2\tau_{O}}\right)\cdot {NBW}_{WSS}$$

## 4. High-Gain Single-Ended Comparator for Digital-Pixel Sensor

### 4.1. Pixel Circuit in the Digital-Pixel Sensor

A large $C_{bw}$ reduces RN by limiting the NBW, but we need to consider the $C_{C}$ value and memory area in the limited area with available devices. Instead of large $C_{bw}$, a large open-loop gain helps reduce TN at T2 in Figure 2, as shown in Equation (5). RN at T0 can be suppressed by increasing $C_{C}$ even with a small $C_{bw}$. A comparable RN to D-CDS is available in this design strategy with a single memory bank for a small-pixel-size DPS.

**Figure 8:**
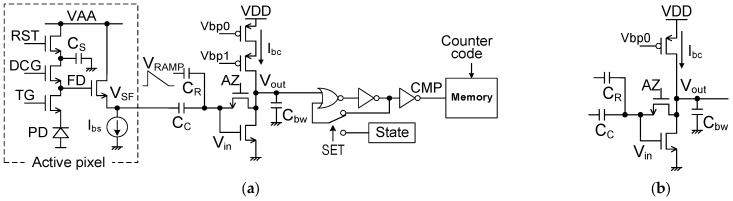
Pixel configuration using a single-ended comparator: (**a**) pixel configuration using a single-ended amplifier with a cascode load transistor (w/cascode); and (**b**) a non-cascode amplifier (w/o cascode).

The single-ended amplifier, instead of the differential amplifier in Figure 1, also helps with the pixel size reduction. The pixel configuration using a single-ended comparator for the 3.24 μm DPS pixel is shown in Figure 8a. It was implemented in one of the test chips, which has the same chip configuration reported in [16], with a couple of variations in comparator circuits and layout modifications. This active pixel has a dual-conversion gain gate (DCG), which enables low conversion gain when it turns on. The pixel memory is 10 bits without D-CDS operation, but the FPN is suppressed by on-chip image signal processing (ISP) using reference frame data with quasi-dark images [16]. The ramp signal for SS-ADC is given through the additional input capacitor $C_{R}$. This comparator structure has two advantages compared to the differential amplifier. The first is design flexibility for small pixel sizes. Its simple configuration enables the use of a relatively large transistor for smaller FN and larger gain. The second is a larger ADC range under lower supply voltage, because the comparator flips at the same input voltage regardless of the pixel signal voltage $V_{SF}$.

Although this single-ended comparator configuration has a disadvantage that the signal is attenuated by the ratio of $C_{C}/(C_{C}\;+\;C_{R})$ at $V_{in}$, it is not critical from the point of view of the S/N ratio. This is because the noise PSD of ${e_{n}}^{2}$ and ${V_{TN}}^{2}$ decreases to half, as shown in Equations (19) and (20), when the single-ended amplifier, instead of the differential amplifier, is used as the comparator input stage. If $C_{C}/(C_{C}\;\;+\;\;C_{R})\;=2/3$, a smaller RN of a single-ended comparator can compensate for most of the signal attenuation and achieve almost the same S/N ratio.

(19) $${e_{n}}^{2}=\frac{4kT\gamma }{g_{mi}}(1+\frac{g_{mo}}{g_{mi}}):\mathrm{Single-ended}\;\mathrm{amplifier}$$

(20) $${V_{TN}}^{2}=\frac{\gamma kT}{G\cdot C_{bw}}\left(1+\frac{g_{mo}}{g_{mi}}\right):\mathrm{at}\;T1,T2\;{V_{TN}}^{2}=\frac{\gamma kT}{C_{C}+C_{bw}}\left(1+\frac{g_{mo}}{g_{mi}}\right):\mathrm{at}\;T0$$

### 4.2. Measurement Results

We evaluated two types of amplifiers in the comparator. The first is the cascode configuration of the pMOS load transistor, as shown in Figure 8a. The open-loop gain of this configuration is G≈190 under the condition of VDD = 1.1 V and $I_{bc}=20\;nA$. The other type is a simplified amplifier, as shown in Figure 8b. The open-loop gain is G≈85 under the same condition.

**Figure 9:**
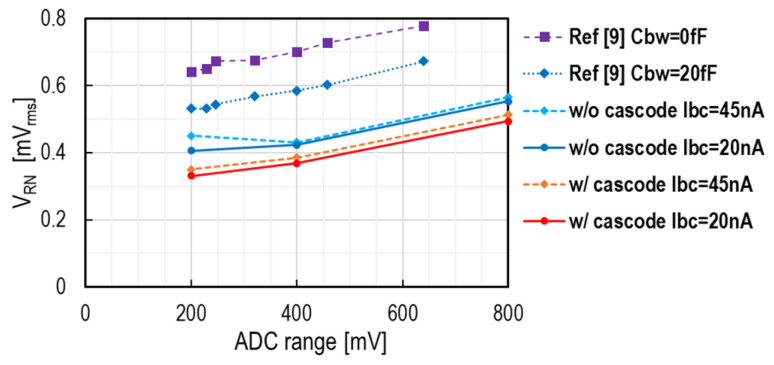
Noise measurement results of the test chip of two types of amplifiers, with that of the reported chip in [9]. SS-ADC operation is 10-bit in this work and 9-bit in Ref. [9].

Design parameters and RN evaluation results are summarized in Table 1. RN voltages at the source follower output ($V_{SF}$) of two types of pixel comparators are also compared in Figure 9. A lower noise voltage than the previous chip is achieved with a smaller $C_{bw}$. Its contributors are the large $C_{C}$ using a high-density 3D-MIM capacitor and the large open-loop gain G of the single-ended amplifier. To achieve a small RN in equivalent electron number, we designed this pixel to have an HCG of 208 μV/e−. Thanks to this HCG, the test chip with the cascode configuration demonstrated 2.2 e-_rms_ RN with an ADC range of 400 mV.

**Figure 10:**
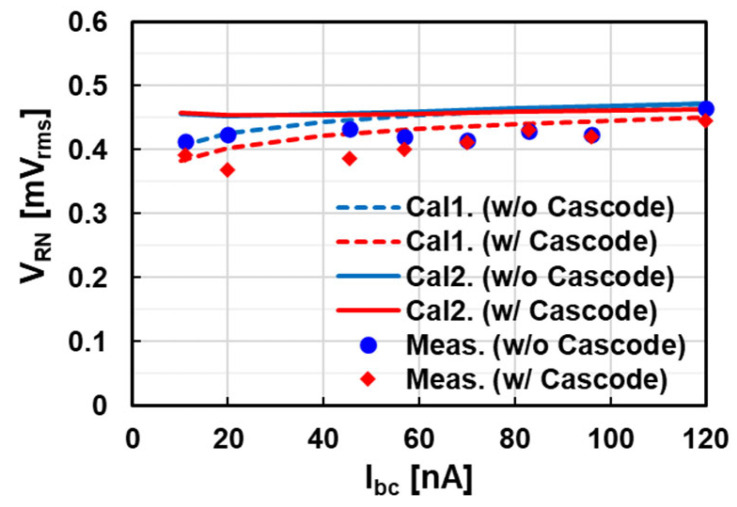
Noise measurement results of two types of pixels in Figure 8 by sweeping bias current and comparison with two calculation results. Cal 1 is a WSS condition, and Cal 2 is a nonstationary condition. SS-ADC operation is 10-bit 400 mV.

The bias current dependencies of RN in the test chips of two types of amplifiers, each available with or without a cascode, are shown in Figure 10 under the condition of VDD = 1.1 V. The measurement results are revised from those under the condition of VDD = 0.97 V reported in [17]. RN becomes small with a reasonable trend.

The calculated RNs under the two conditions of WSS (Cal1) and nonstationary (Cal2) for two types of amplifiers are plotted in Figure 10 to compare with the measurement results. RN voltages under the WSS condition are calculated as follows.

(1) *G*, $g_{mi}$, and ${{e_{n}}^{2}f}_{CN}$ at each bias current are measured using SPICE simulation.
(2) Each TN ($V_{TN})$ at T0 and T2 is calculated using Equation (20).
(3) Each FN ($V_{FN})$ at T0 and T2 is calculated using $f_{CO}$ of Equation (5).
(4) Each RN ($V_{RN})$ at T0 and T2 is calculated using $\sqrt{{V_{TN}}^{2}+{V_{FN}}^{2}}$.
(5) The total input-referred RN at V_SF_ is calculated by $(C_{C}+C_{R})/C_{C}\sqrt{{V_{RN(T0)}}^{2}+{V_{RN(T2)}}^{2}}$ using the RN at T0 and T2 with compensation of signal attenuation from V_SF_ to V_in_.

RN voltages under the nonstationary condition are also calculated in the same manner, assuming an effective NBW as $f_{CO}^{’}=\;K_{bw}\;\cdot \;f_{CO}$*,* where $K_{bw}$ is calculated from $t_{0}/\tau_{O}$ using Equations (17) and (18).

The calculated RN values roughly match the measurement results. The difference between the two calculation methods is small because the main contributor is FN, which is less sensitive to the NBW. The breakdowns of TN and FN in each RN are shown in Section 4.3.

### 4.3. Noise Analysis of the Single-Ended Comparator

**Figure 11:**
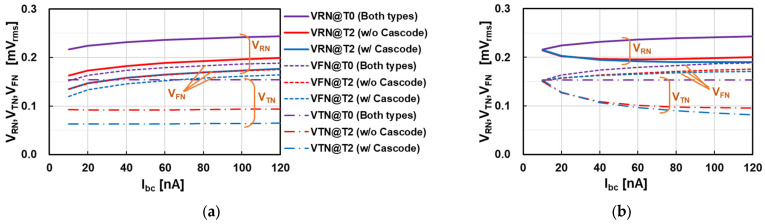
Calculated input-referred RN (*V_RN_*) in the single-ended amplifier associated with TN (*V_TN_*) and FN (*V_FN_*): (**a**) conventional AC noise estimation under the WSS condition; (**b**) nonstationary condition after effective NBW compensation by the coefficient of *K_BW_*.

Figure 11 shows the calculation results of RN ($V_{RN}$), TN ($V_{TN}$), and FN ($V_{FN}$) at $V_{in}$ in the single-ended comparator in Figure 8. Two amplifier types (w/o cascode and w/cascode) were assumed to calculate the noise. However, the noise voltages at T0 become identical because of the closed-loop configuration, so the two plots at T0 are merged. The gain difference between the two amplifier types gives the impact of those at T2 for comparator operation. Figure 11a shows the calculation results under the WSS condition. The FN difference between the three plots is relatively small, while a large difference appears in TN. The calculation results show that a large gain helps reduce TN, and FN becomes the main contributor, especially in the large-gain case (w/cascode).

Figure 11b shows the calculated RN with TN and FN under the nonstationary condition. Those at T0 are the same as those in Figure 11a because of the non-comparator operation. Those at T2 are impacted by the ramp slope. The effective NBW is assumed to be $K_{bw}$ times larger, as calculated by $t_{0}/\tau_{O}$ and shown in Figure 7b. Even if the ramp slope is fixed, a small bias current $I_{bc}$ (small $g_{mi}$) makes $K_{bw}$ large by the small $t_{0}/\tau_{O}$. Therefore, noise voltages at T2 increase as $I_{bc}$ decreases. A larger *G* of the cascode amplifier makes $t_{0}/\tau_{O}$ small, and the noise voltage in the cascode amplifier increases more than that of the amplifier without the cascode configuration, resulting in noise differences between the two amplifier configurations being small under the nonstationary condition. Increased RN at T2 approaches the RN at T0 in the small $I_{bc}$ region, because $K_{bw}$ increases as $I_{bc}$ decreases. This means that the effectiveness of RN reduction in D-CDS operation becomes small in small-bias cases. The calculation result shown in Figure 11b indicates that FN is the main contributor of RN, especially in the large-bias current region. It also suggests that the comparator bias current and the ramp slope should be carefully chosen for a smaller TN.

The test chips showed better RN performance than the previous chips [9] by limiting the NBW with a higher gain. However, for further RN improvement, FN reduction for the input stage amplifier of the comparator should be prioritized because the limiting NBW for RN reduction is inefficient under the large FN case.

## 5. Conclusions

RN estimation for SS-ADC in small pixel DPSs has been discussed in both WSS and nonstationary conditions. We have shown two convenient equations that make the SS-ADC design efficient. One is the FN/TN ratio under the WSS condition. The other is a concept of the slope factor ($t_{0}/\tau_{O}),$ which leads to a simple estimation of the effective NBW. FN can become the main contributor to RN with a relatively small band-limiting capacitance $C_{bw}$ in the comparator. RN reduction by D-CDS with the limiting NBW is not an efficient solution for a small-pixel DPS that requires doubled in-pixel memory area, as far as FN is the primary contributor to RN. An improved RN performance of 2.2 e-_rms_ has been demonstrated with a high-gain single-ended comparator and small $C_{bw}$ in a 3.24 μm pixel without D-CDS. Noise analysis suggests that FN improvement is the highest priority for further noise improvement. The CMS is one of the possible resolutions if an effective circuit scheme is considered.

## Figures and Tables

**Table 1:** Design parameters and RN performance comparison with the previous chip.

Specification	This Work	TED 2022 [9]
Process technology	45/40/40 nm	45/65 nm
Pixel size [µm]	3.24	4.6
Conversion Gain [µV/e−]	208/19.4	170/7
Comparator type	Single-ended input	Differential input
C_C_ [fF]	120	65
C_R_ [fF]	60	-
C_bw_ [fF]	6	20
RN [e-_rms_]	2.2	4.2

## Data Availability

The original contributions presented in this study are included in the article. Further inquiries can be directed to the corresponding author.

## Abbreviations

V_RN_	Input-referred RN
V_TN_	Input-referred TN
V_FN_	Input-referred FN
k	$\mathrm{Boltzmann}\;\mathrm{constant}\;(1.38\;\times 10^{-23}$)
T	Temperature (300 is used for calculation)
γ	Body-effect parameter (2/3 is used for calculation)
$e_{n}$	PSD of TN
G	Open-loop gain of comparator 1st stage
$g_{mi}$	Transconductance of input MOS transistor
$g_{mo}$	Transconductance of load MOS transistor
$f_{CN}$	Corner frequency (TN PSD = FN PSD)
$f_{CO}$	Cut-off frequency
$f_{sp}$	Sampling frequency in CDS operation
$f_{0dB}$	Unity gain frequency
$\tau_{O}$	Amplifier time constant
$K_{RAMP}$	Input ramp signal slope
t_d_	Comparator delay time
$K_{bw}$	Effective NBW coefficient
